# On Possible Electromagnetic Precursors to a Significant Earthquake (Mw = 6.3) Occurred in Lesvos (Greece) on 12 June 2017

**DOI:** 10.3390/e21030241

**Published:** 2019-03-02

**Authors:** Stelios M. Potirakis, Alexander Schekotov, Yiannis Contoyiannis, Georgios Balasis, Grigorios E. Koulouras, Nikolaos S. Melis, Adamantia Zoe Boutsi, Masashi Hayakawa, Konstantinos Eftaxias, Constantinos Nomicos

**Affiliations:** 1 Department of Electrical and Electronics Engineering, University of West Attica, Campus 2, 250 Thivon and P. Ralli, Aigaleo, GR-12244 Athens, Greece; 2 Institute of Physics of the Earth, Russian Academy of Sciences, 10 Bolshaya Gruzinskaya, Moscow 123995, Russia; 3 Institute of Astronomy, Astrophysics, Space Applications and Remote Sensing, National Observatory of Athens, Metaxa and Vasileos Pavlou, Penteli, GR-15236 Athens, Greece; 4 Institute of Geodynamics, National Observatory of Athens, Lofos Nimfon, Thissio, GR-11810 Athens, Greece; 5 Hayakawa Institute of Seismo Electromagnetics, Co.Ltd. (Hi-SEM), UEC Alliance Center #521, 1-1- Kojima-cho, Chofu-shi, Tokyo 182-0026, Japan; 6 University of Electro-Communications, Advanced Wireless & Communications research Center (AWCC), Chofu, Tokyo 182-8585, Japan; 7 Department of Physics, Section of Solid State Physics, University of Athens, Panepistimiopolis, GR-15784, Zografos, GR-15784 Athens, Greece

**Keywords:** The 2017 Lesvos earthquake (EQ), ultra-low-frequency (ULF) effects, EQ precursor, lithospheric ULF emission, ULF depression, fracto-electromagnetic emissions

## Abstract

This paper reports an attempt to use ultra-low-frequency (ULF) magnetic field data from a space weather monitoring magnetometer array in the study of earthquake (EQ) precursors in Greece. The data from four magnetometer stations of the Hell*ENI*c *G*eo*M*agnetic *A*rray (*ENIGMA*) have been analyzed in the search for possible precursors to a strong EQ that occurred south of Lesvos Island on 12 June 2017, with magnitude Mw = 6.3 and focal depth = 12 km. The analysis includes conventional statistical methods, as well as criticality analysis, using two independent methods, the natural time (NT) method and the method of critical fluctuations (MCF). In terms of conventional statistical methods, it is found that the most convincing ULF precursor was observed in the data of ULF (20–30 mHz) depression (depression of the horizontal component of the magnetic field), which is indicative of lower ionospheric perturbation just 1 day before the EQ. Additionally, there are indications of a precursor in the direct ULF emission from the lithosphere 4 days to 1 day before the EQ. Further study in terms of NT analysis identifies criticality characteristics from 8 to 2 days before the EQ both for lithospheric ULF emission and ULF depression, while MCF reveals indications of criticality in all recorded magnetic field components, extending from 10 to 3 days before the EQ. Beyond the recordings of the geomagnetic stations of ENIGMA, the recordings of the fracto-electromagnetic emission stations of the H*EL*lenic *S*eismo-*E*lectro*M*agnetics *Net*work (*ELSEM-Net*) in Greece have been analyzed. The MHz recordings at the station that is located on Lesvos Island presented criticality characteristics (by means of both NT analysis and MCF) 11 days before the EQ, while a few days later (7–6 days before the EQ), the kHz recordings of the same station presented tricritical behavior. It is noted that the magnetosphere was quiet for a period of two weeks before the EQ and including its occurrence.

## 1. Introduction

There has been a significant progress in the field of electromagnetic (EM) phenomena associated with earthquakes (EQs), so-called seismo-electromagnetics, during the last few decades, and it is recently agreed that EM effects take place not only in the lithosphere, but also in the atmosphere and ionosphere before a major EQ [1,2,3,4]. The direct effect of EQs is EM emission in a wide frequency range from direct current (DC) to very high frequency (VHF) (or even higher), and the indirect effects of EQs are seismo-atmospheric and -ionospheric perturbations that are detected with the use of radio techniques, while one of the main objectives of seismo-electromagnetics is the elucidation of the lithosphere-atmosphere-ionosphere (LAI) coupling process, which is still controversial (e.g., [4]).

It is well known that the continuous monitoring of ultra-low frequency (ULF) (≤1 Hz) geoelectric signals (Seismic Electric Signals, SES) is in extensive operation for a long time in Greece (e.g., [5,6]), and that MHz-kHz fracto-electromagnetic emissions (fracto-EME or simply EME) are also being observed in Greece for long time [7,8,9,10]. Moreover, it has been reported that magnetic field variations accompany the appearance of SES signals [8]. However, to the best of our knowledge, an analysis of ULF magnetic field data from a space weather monitoring fluxgate magnetometer array within the framework of seismo-electromagnetics or EQ prediction studies has never been carried out in Greece, even though ULF magnetic field variations from such magnetometer arrays have been found to be of great importance in EQ precursor studies for short-term EQ prediction [2,3].

There are two types of anomalies in the ULF effects in possible association with EQs. The first is the well-known conventional ULF direct lithospheric emissions, which have been considered to be an important candidate of EQ precursors for a long time, since the 1988 Spitak EQ [11,12,13,14], reflecting the generation of electric currents due to the pre-EQ fracturing process in the lithosphere. On the other hand, the second is a rather recent phenomenon, named ULF depression, which appeared in the form of depression (decrease, reduction) of the horizontal component of the magnetospheric ULF Alfvén waves that were observed on the ground, which can be attributed to the changes in the lower ionosphere [15,16,17].

In the present article, we focus on the above-mentioned two types of ULF anomalies, as well as on MHz and kHz fracto-EME signals that were recorded prior to a rather strong EQ of magnitude Mw = 6.3, which occurred in Greece (south of Lesvos Island) on 12 June 2017 at 12:28:39 UT in a focal depth of 12 km. We find that multiple evidence corroborate the view that the specific EQ came with EM precursors.

Specifically, we first apply conventional statistical analysis to specifically defined daily-valued ULF quantities [15,16,17,18], as calculated from the magnetic field measurements of four magnetometer stations that are scattered around Greece. From this analysis, we find notable anomalies in the ULF depression, which are indicative of the lower ionospheric perturbation, at two stations, at the station closest to the epicenter and a more distant station, one day before the EQ. Moreover, there are indications of a precursor in the direct ULF emission from the lithosphere four days to one day before the EQ at the same two stations. In further investigating magnetometer data, we apply the natural time (NT) criticality analysis method [19] to the above-mentioned daily-valued ULF quantities for the two stations for which possible precursors to the EQ were identified. NT analysis identifies the criticality characteristics from eight to two days before the EQ both for lithospheric ULF emission and ULF depression. Additionally, we apply another criticality analysis method, referred to as the method of critical fluctuations (MCF) [20,21,22,23] to the raw (unprocessed) magnetometer recordings, revealing indications of criticality in all recorded magnetic field components, extending from ten to three days before the EQ.

Since this is probably the first attempt in Greece to use ULF magnetic field data from a space weather monitoring fluxgate magnetometer array in the study of EQ precursors, we analyzed another type of EM signals possibly related to EQs to check whether these also contain anomalies during the period that the ULF anomalies were identified. Indeed, by analyzing the data from a ground-based fracto-EME station network that spans across Greece, we find that the MHz recordings at the station located on Lesvos Island presented criticality characteristics (both by means of NT analysis and MCF) 11 days before the EQ, while a few days later (7–6 days before the EQ) the kHz recordings of the same station presented tricritical behavior.

## 2. Electromagnetic Observatories and Earthquake Information

**Figure 1:**
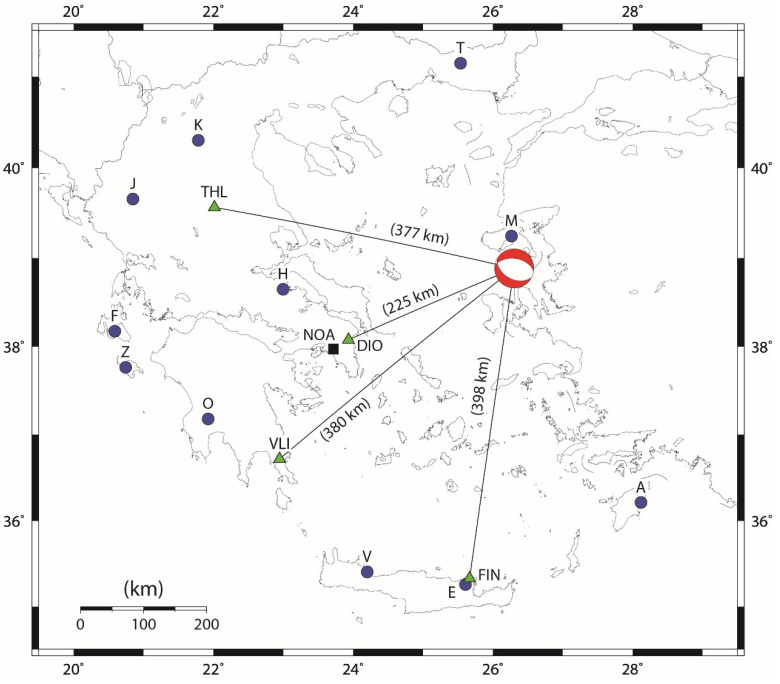
Map showing the 4 magnetometer stations of ENIGMA (green triangles) and the 11 fracto-electromagnetic emissions (fracto-EME) stations of the ELSEM-Net (blue circles) in Greece. All of the stations are linked to Institute of Geodynamics of the National Observatory of Athens (NOA-IG) in Athens (black square). The 2017 Lesvos earthquake (EQ) is noted with the moment tensor (MT) solution computed by NOA-IG (see http://bbnet.gein.noa.gr/mt_solution/2017/170612_12_28_38.00_MTsol.html). No other strong EQs (Mw > 5.5) occurred in Greece during the time period 1 May 2017–17 June 2017. (For interpretation of the references to colors, the reader is referred to the online version of this paper.).

A strong EQ of magnitude Mw = 6.3 occurred in Greece on 12 June 2017 at 12:28:39 UT. According to National Observatory of Athens, Institute of Geodynamics, (NOA-IG), the EQ epicenter was located at the geographic coordinates (38.930° N, 26.365° E), 5 km south of town Plomari of Lesvos Island, as shown in Figure 1, with a focal mechanism solution (see http://bbnet.gein.noa.gr/mt_solution/2017/170612_12_28_38.00_MTsol.html) presented, and its focal depth was 12 km.

The *ENIGMA* (Hell*ENI*c *G*eo*M*agnetic *A*rray) [http://enigma.space.noa.gr/] ground-based fluxgate magnetometer network, as shown in Figure 1 by green triangles, acquired the geomagnetic data that were used in this paper. ENIGMA, which is operated by the National Observatory of Athens (NOA), is the first and it presented only permanent magnetometer network that ever operated in Greece and within a few years of operations achieved becoming a member of SuperMAG [http://supermag.jhuapl.edu/]. The array consists of four ground-based magnetometer stations in the areas of Trikala (Klokotos—THL), Attica (Dionysos—DIO), Lakonia (Velies—VLI), and Lasithi (Finokalia—FIN) in Greece that primarily targeted providing measurements for the study of geomagnetic pulsations, resulting from the solar wind-magnetosphere coupling. The respective coordinates, altitude, and instrumentation of the stations are summarized in Table 1. During the period of interest (1 May 2017–17 June 2017), ULF geomagnetic field data from all four magnetometer stations were available. The ULF data with a sampling frequency higher than 1 Hz were downsampled to 1 Hz.

Ground-based magnetometers have proven to be the workhorse of magnetosphere-ionosphere coupling physics. The instruments that were used are survey vector (fluxgate) magnetometers, which means that three orthogonal sensors are required to measure the components of the magnetic field in all three dimensions, along with their fluctuations. ENIGMA measures the geomagnetic field in magnetic coordinates (i.e., H and D represent horizontal variations, while Z vertical variations of Earth’s magnetic field). The ENIGMA magnetometer array enables the effective remote sensing of geospace dynamics and the study of space weather effects on the ground (i.e., Geomagnetically Induced Currents—GIC). ENIGMA contributes data to SuperMAG, which provides easy access to validated ground magnetic field perturbations in the same coordinate system, identical time resolution, and with a common baseline removal approach. The purpose of SuperMAG is to help scientists, teachers, students, and the general public have easy access to measurements of the Earth’s magnetic field [24].

The here employed MHz and kHz signals were recorded by the *ELSEM-Net* (H*EL*lenic *S*eismo-*E*lectro*M*agnetics *Net*work) (http://elsem-net.uniwa.gr) ground-based fracto-EME station network, spanning across Greece, as shown in Figure 1 by blue circles (see also Table 2). Specifically, these were recorded at the Agia Paraskevi, Lesvos (M) station. The EM variations that are continuously monitored by ELSEM-Net at the Zante (Z) station correspond to narrowband (Q > 10) 3 kHz north–south, 3 kHz east–west, 3 kHz vertical, 10 kHz north–south, 10 kHz east–west, and 10 kHz vertical magnetic field, as well as 41, 54, and 135 MHz electric field recordings, while at the rest of the stations, including the Agia Paraskevi, Lesvos station, only the 3 kHz north–south, 3 kHz east–west, 10 kHz north–south, 10 kHz east–west magnetic, and 41, 46 MHz electric field are recorded. Both the kHz and MHz receivers are custom-made, providing, as outputs, the RMS values of kHz and the signal strength of MHz signals, after a <0.5 Hz low pass filtering. All of the receiver outputs are sampled at $f_{s}=1\;\mathrm{Hz}$. More details on the instrumentation of the fracto-EME stations of ELSEM-Net can be found in the supplementary downloadable material of [9].

## 3. Possible ULF EQ Precursors by Means of Conventional Statistical Analysis

In the following we try to detect any possible EQ related ULF anomalies by means of conventional statistical analysis. This can be defined as a method to identify any extreme values, i.e., local maxima or local minima of specific ULF quantities [15,16,17,18].

**Figure 2:**
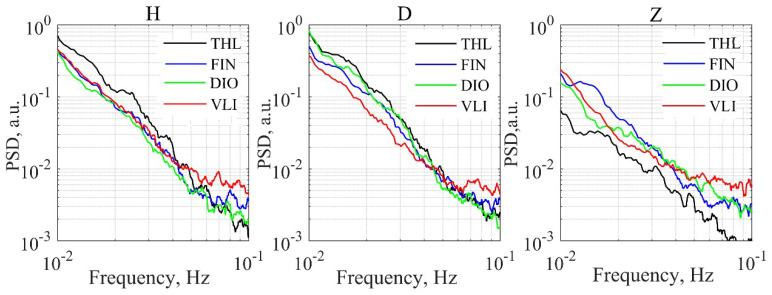
Averaged night background spectra of H, D, and Z components for all magnetometers. (For interpretation of the references to colors, the reader is referred to the online version of this paper).

As the initial step of the ULF conventional statistical analysis, we have estimated the averaged night (00 h–04 h, UT) background spectra of H, D, and Z components for all magnetometers, after it was found that industrial interferences at this time interval are minimal. Figure 2 shows these spectra in the frequency band 10–100 mHz, averaged over the time period from 1 May to 17 June 2017, from which one observes that the spectra of the horizontal components are about the same. The background level is only slightly higher in THL. As expected, the vertical components present lower power spectral density (PSD) values when compared to the horizontal components.

Based on these preliminary analysis results, we decided to try to detect any EQ precursors by using ULF data from all four stations: the closest to the EQ epicenter DIO as well as the other more distant stations to investigate the distance effect. Note that DIO is located at about 250 km, while the other three stations are located at about 400 km from the EQ epicenter (see Figure 1).

**Figure 3:**
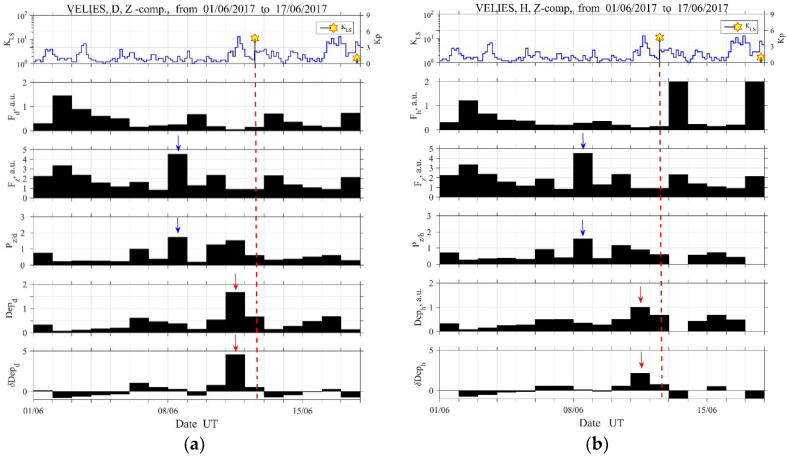
Analysis results for a short period of 1–17 June 2017 at Velies (VLI) for D, Z components (**a**) and H, Z components (**b**). In each figure, top panel indicates the temporal evolutions of geomagnetic activity (Kp) and seismic activity ($K_{LS}$). The second and third panels refer to the mean power of the horizontal ($F_{d}$ or $F_{h}$) and vertical components ($F_{z}$), and the fourth, to their ratio (polarization) ($P_{z/d}$ or $P_{z/h}$). The fifth panel refers to the inverse of the mean power of the horizontal component (the horizontal depression, $Dep_{d}$ or $Dep_{h}$) and the bottom one to its relative change ($\delta Dep_{d}$ or $\delta Dep_{h}$). Vertical dashed lines mark the time of occurrence of the 2017 Lesvos EQ. (For the interpretation of the references to colors, the reader is referred to the online version of this paper).

**Figure 4:**
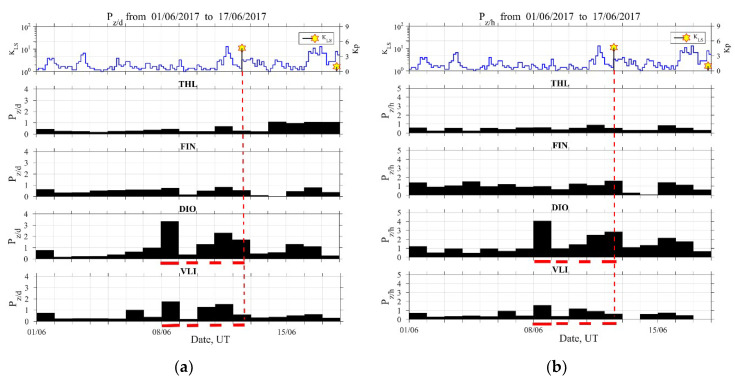
Evolution of the lithospheric ultra-low frequency (ULF) quantities $P_{z/d}$ (a) and $P_{z/h}$ (b) in all four observation sites. Top panel is similar to the top panel of Figure 3. Vertical dashed lines mark the time of occurrence of the 2017 Lesvos EQ. Horizontal dashed lines mark a noticeable increase of these quantities at the nearest DIO station, which is also present, although at a lower level, in the distant station VLI.

**Figure 5:**
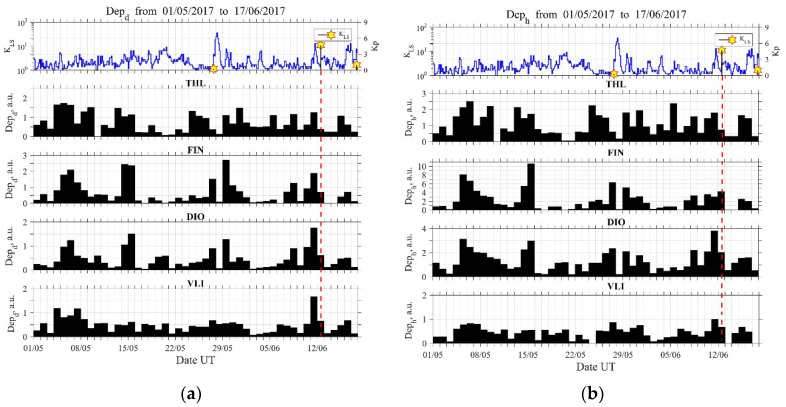
Evolution of the ionospheric ULF quantity absolute depression for the D (**a**) and H (**b**) horizontal components in all four stations. Top panel is similar to the top panel of Figure 3. Vertical dashed lines mark the time of occurrence of the 2017 Lesvos EQ. The most profound anomaly is observed for D component at VLI.

**Figure 6:**
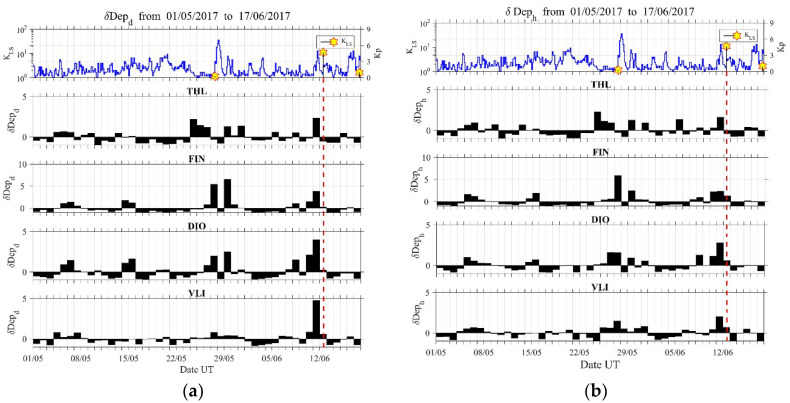
Evolution of the ionospheric ULF quantity relative depression for the D (**a**) and H (**b**) horizontal components in all four observation sites. Top panel is similar to the top panel of Figure 3. Vertical dashed lines mark the time of occurrence of the 2017 Lesvos EQ. The most profound anomaly is observed for D component at VLI.

When trying to detect possible ULF EQ precursors, we have to plot the temporal evolutions of geomagnetic and seismic activities in parallel to the studied ULF quantities, because we have to distinguish between the seismic and space weather effects. This information is plotted in the top panels of Figure 3, Figure 4, Figure 5 and Figure 6, in which the Kp index indicates the geomagnetic activity and $K_{LS}$ (local seismicity) is the seismic index defined by $K_{LS}=10^{0.75M}/\left(R+100\right)$ (R: epicentral distance in km, M: EQ magnitude) [2,15,16]. Since a $K_{LS}$ value higher than 1 usually means a significant EQ that could have precursors, this 2017 Lesvos EQ is found to be a remarkable event.

In each one of Figure 3a,b, we plot a number of different ULF quantities for a 17-days-long, period starting from 1 June 2017, as calculated from the D, Z (Figure 3a) and the H, Z (Figure 3b) magnetic field components that were observed at VLI. These are the mean power of a horizontal magnetic field component (D or H) in the second panel, $F_{d}=\langle D_{\Delta T,\Delta f}^{2}\rangle$ (Figure 3a) or $F_{h}=\langle H_{\Delta T,\Delta f}^{2}\rangle$ (Figure 3b), the mean power of the vertical magnetic field component in the third panel, $F_{z}=\langle Z_{\Delta T,\Delta f}^{2}\rangle$, and the ratio of vertical to horizontal mean power in the fourth panel, $P_{z/d}=F_{z}/F_{d}$ (Figure 3a) or $P_{z/h}=F_{z}/F_{h}$ (Figure 3b), [15,16,17,18]. In the fifth and sixth panel, respectively, we plot the absolute, $Dep_{d}$ (Figure 3a) or $Dep_{h}$ (Figure 3b), and the relative, $\delta Dep_{d}$ (Figure 3a) or $\delta Dep_{h}$ (Figure 3b), ULF depression (defined later) [15,16]. The above defined ULF quantities are estimated in a specific frequency band (Δf) averaged over the (local) midnight interval (ΔT).

The quantities portrayed in the third and fourth panel in Figure 3a,b are considered to be a possible indicator of conventional lithospheric emission (e.g., [13,14,15,16,17]). While, the bottom two refer to the inverse of the horizontal component, $Dep_{d}$ or $Dep_{h}$, and its relative variation, $\delta Dep_{d}$ or $\delta Dep_{h}$. These two quantities are not so common among the scientific society and they need some explanation [15]. These indicate the depression (decrease, reduction) of the horizontal component (mean power) of ground-based ULF magnetic field. The value of absolute depression of the horizontal component is calculated as the inverse of the horizontal component mean power, $Dep_{d}=1/F_{d}$ or $Dep_{h}=1/F_{h}$ for the D or H component, respectively. On the other hand, the relative change in depression for the i-th day, is calculated as $\delta Dep_{d}=\left(Dep_{d,i}-\frac{1}{N}\sum_{j=i-N}^{i-1}Dep_{d,j}\right)/\left(\frac{1}{N}\sum_{j=i-N}^{i-1}Dep_{d,j}\right)$ or $\delta Dep_{h}=\left(Dep_{h,i}-\frac{1}{N}\sum_{j=i-N}^{i-1}Dep_{h,j}\right)/\left(\frac{1}{N}\sum_{j=i-N}^{i-1}Dep_{h,j}\right)$ for the D or H component, respectively, where N is equal to the number of preceding days for averaging. The denominator indicates the average value and the numerator means the deviation from the mean. The above defined ULF quantities are known to indicate the absorption (or scattering) of downgoing Alfvén waves from the magnetosphere [2,17], and which is quite similar in nature to the lower ionospheric perturbations as detected by subionospheric VLF propagation anomalies [25,26]. Additionally, the statistical former result [15] indicated that the values of Dep and δDep are proportional to the magnitude of an imminent EQ.

Based on the analysis of the EM background (see, for example, Figure 2), we have adopted a time window (ΔT) of 00–04 h UT (i.e., 02–06 h LT) with the lowest interferences, frequency band (Δf) of 20–30 mHz as the most remarkable band, and an averaging interval of N=10 days for $\delta Dep_{d}$ and $\delta Dep_{h}$.

Figure 3 suggests very clear enhancements in $Dep_{d}$ and $Dep_{h}$, as well as in $\delta Dep_{d}$ and $\delta Dep_{h}$ one day before the EQ (marked by red arrows). This is especially visible in D component (Figure 3a). Moreover, the ratio of the vertical to horizontal mean power as the most important parameter for ULF lithospheric emission [18] shows an enhancement from four days (marked by blue arrows) to one day before the EQ for both horizontal components ($P_{z/d}$, $P_{z/h}$).

Focusing on the possible lithospheric ULF emission, Figure 4 shows the temporal evolution of mean power ratios $P_{z/d}$ (Figure 4a) and $P_{z/h}$ (Figure 4b) in all stations for the same time period as in Figure 3. This ULF quantity presents a remarkable increase in the data of both components for the nearest station DIO for four days before the EQ, while this increase, though noticeably less in level, is also observed at a more distant point in VLI. This indicates a probable observation of lithospheric signal. At the same time, this precursor was not detected in the rest distant stations, FIN and THL.

In more detail, it can be seen from Figure 4 at the station DIO closest to the EQ epicenter (distance about 250 km) that, during the whole period, there exists a significant enhancement, a double-peaked broad maximum from four days to one day before the EQ. On the other hand, for the farther station of VLI, we may find the corresponding maximum at this station as well, although at lower levels. As for the peak appearing one day before the EQ, it is possible that this enhancement in $P_{z/d}$ and $P_{z/h}$ may be related with the decrease in $F_{d}$ and $F_{h}$ (i.e., enhancement in $Dep_{d}$ and $Dep_{h}$) or local interferences on this day. However, the peak in both mean power ratios four days before the EQ is highly likely to be a precursor to this EQ. This ULF emission is a local effect and the previous works on ULF emission indicate that the nominal detection range is less than 100 km for the present EQ magnitude [13,14]. However, these results suggest that this effect is also likely to be observed over long distances.

In order to further investigate for possible precursors, we have plotted the temporal evolutions of the ionospheric ULF quantities, absolute (Figure 5) and relative depression (Figure 6), for a much longer time period (about 1.5 months) from 1 May 2017 to 17 June 2017 for all sites and both of the horizontal components. One can observe that the most reliable detection of an anomaly, in both quantities of depression, is obtained for the D component that was observed at VLI, while much less reliable results are obtained for the other sites.

Specifically, a clear enhancement in $Dep_{d}$ (Figure 5a) and the simultaneous very clear enhancement in $\delta Dep_{d}$ (Figure 6a) (i.e., maximum depression of the horizontal component) can be identified just one day before the EQ mainly at the VLI and DIO stations. The station of VLI is about 400 km away from the EQ epicenter (see Figure 1), but this effect is most clearly noticed there. It is well known from our previous statistical and event studies that this ULF depression effect can be sensitively detected even at far distances from the EQ epicenter [15,16]. Of course, the EQ preparation zone may extend up to ~500 km (in radius) for this EQ magnitude [1,2]. It also has to be noted that the region of depression seldom is exactly above EQ epicenter. One cannot easily define the location of the region in the ionosphere that causes a depression. It is expected to be located over a place of geochemical changes, such as gas emanation. In order to suggest such a region, one should carefully combine information concerning the tectonics of Greece, as well as possible information regarding gas emanation observations. However, this is considered to be out of scope for the present work.

At this point, we have to comment on the important issue of the possible association of the above identified precursors with any space weather activity. The ENIGMA magnetometer array is, in general, orientated towards the study of space weather and extreme space weather events, such as magnetic storms. Nevertheless, during periods of low geomagnetic activity, its recordings can be utilized, as happens with other magnetometers around the world, for the tracking of signals of different frequencies, such as the ones that are discussed in the present study in order to detect the possible precursors of seismic activity. During the period of interest (1 May 2017–17 June 2017), the space weather conditions, and consequently the geomagnetic activity, were extremely low. The only exception was marked on the 28 May 2017 (15 days before the studied EQ), when a moderate geomagnetic storm took place. On that day, the Dst index (http://wdc.kugi.kyoto-u.ac.jp/dstdir/index.html), a proxy of the strength of the magnetospheric ring current, and thus an indicator of magnetic storm intensity lowered to −125 nT at 08:00 UT. This is also captured by the maximum value of the Kp index, as shown in the top panels of Figure 3, Figure 4, Figure 5 and Figure 6. Clearly, this geomagnetic activity event precedes the identified precursors both in ULF depression one day before the EQ and ULF lithospheric emission 4–1 days before the EQ, and for this reason, these are unlikely to be of global origin (space-sourced disturbances). By means of Kp index, one may also find a simultaneous geomagnetic activity one day before the EQ, but this geomagnetic activity is too small to affect our conclusion (possibly just slightly reducing the ULF depression).

## 4. Possible ULF EQ Precursors by Means of Criticality Analysis

In the following, we try to identify any indications of critical dynamics in the ULF magnetic field data by means of two independent time series analysis methods that are known for their ability to uncover critical dynamics. These are the recently proposed methods referred to as the natural time (NT) analysis [19] and the method of critical fluctuations (MCF) [20,21,22,23], as briefly described in Section 4.1 and Section 4.3, respectively. Both of them have been applied to ULF magnetic field data recorded prior to a number of EQs that occurred in Japan, revealing useful information. The first one is applied to the daily valued ULF quantities, as defined in Section 3 [27,28,29,30], while the second one is applied to the raw (unfiltered) magnetometer measurements [31,32].

### 4.1. Natural Time Analysis

The transformation of a time-series of “events” from the conventional time domain to the NT domain is performed by ignoring the time interval between events and only retaining its normalized order of occurrence [19]. Accordingly, the k-th event corresponds to a NT $\chi_{k}=k/N$, where N is the total number of successive events. The “energy” $Q_{k}$ of each event is retained. Subsequently, the transformed time series ($\chi_{k}$, $Q_{k}$) is studied. By defining $p_{k}=Q_{k}/\sum_{n=1}^{N}Q_{n}$, the energy of k-th event normalized by the total energy, a system is considered to approach criticality when the parameter $\kappa_{1}=\sum_{k=1}^{N}p_{k}\chi_{k}^{2}-{\left(\sum_{k=1}^{N}p_{k}\chi_{k}\right)}^{2}$ converges to the value $\kappa_{1}=0.070$, and at the same time, both the entropy in NT, $S_{nt}=\sum_{k=1}^{N}p_{k}\chi_{k}\ln\chi_{k}-\left(\sum_{k=1}^{N}p_{k}\chi_{k}\right)\ln\left(\sum_{k=1}^{N}p_{k}\chi_{k}\right)$ and the entropy under time reversal, $S_{nt-}$, satisfy the condition $S_{nt},S_{nt-}<S_{u}=\left(\ln2/2\right)-1/4\left(\approx 0.0966\right)$, where $S_{u}$ stands for the entropy of a “uniform” distribution in NT [19,33]. This is the set of criteria for criticality, according to NT analysis, which is usually applied to time series [19], and it has been applied to different raw (unprocessed) EM recordings that are possibly related to EQ, such as the ultra-low frequency (≤1 Hz), Seismic Electric Signals (SES) [5,34,35,36], and the fracto-EME MHz signals [8,9,10].

Moreover, NT analysis is also applied to daily-valued quantities, such as the ULF magnetic field quantities that are defined in Section 3 [27,28,29,37], and VLF subionospheric propagation quantities [30], although in such cases there is usually a limited number of available data, as happens with the NT analysis of foreshock seismicity. However, in such cases, criticality is checked differently following the paradigm of NT analysis of foreshock seismicity [33,34,36,38].

1. Specifically, the temporal evolution of specific NT analysis parameters is studied by progressively including new events in the analysis and each time calculating $\kappa_{1}$, $S_{nt}$, $S_{nt-}$, and 〈D〉, based on the already included events. Note that 〈D〉 is the “average” distance $\langle D\rangle =\langle \left|\Pi \left(\varpi \right)-\Pi_{critical}\left(\varpi \right)\right|\rangle$ between the curves of normalized power spectra $\Pi \left(\varpi \right)={\left|\sum_{k=1}^{N}p_{k}\exp\left(j\varpi \chi_{k}\right)\right|}^{2}$ (ϖ is the natural angular frequency, ϖ=2πφ, with φ standing for the frequency in NT, termed “natural frequency”) of the evolving seismicity and the theoretical estimation of Π(ϖ) for $\kappa_{1}=0.070$, $\Pi_{critical}\left(\varpi \right)\approx 1-\kappa_{1}\varpi^{2}$ [19]. Of course, in each step the time series, ($\chi_{k}$, $Q_{k}$) is rescaled in the NT domain, since each time the $k^{th}$ event corresponds to a NT $\chi_{k}=k/N$, where N is the progressively increasing total number of the considered successive events; then, all of the parameters involved in the NT analysis are calculated for this new time series; this process continues until the time of occurrence of the main EQ event. In the resultant time evolution of $\kappa_{1}$, $S_{nt}$, $S_{nt-}$, and 〈D〉, criticality is considered to be truly achieved when, at the same time [19,34]: (i) $\kappa_{1}$ approaches $\kappa_{1}=0.070$ “by descending from above”, (ii) $S_{nt},S_{nt-}<S_{u}$, (iii) $\langle D\rangle <10^{-2}$, and (iv) since the underlying process is expected to be self-similar, the time of criticality does not significantly change by varying the “magnitude” threshold. Although the selection of thresholds is arbitrary in the case of the ULF magnetic field and VLF subionospheric propagation quantities (usually more than 20 threshold values equispaced between zero and a maximum threshold value that is larger than the 50% of the maximum value of the examined quantity are considered), if criticality conditions are met in close dates for more than one of the considered threshold values, and then this is considered to be an indication of the validity of the performed analysis. It may be worth mentioning that these criticality conditions, including $\kappa_{1}=0.070$, were not derived theoretically, but rather empirically from computing the $\kappa_{1}$ values for well-known phenomena and the actual data of seismicity in Greece.

### 4.2. NT Analysis of ULF Data

**Figure 7:**
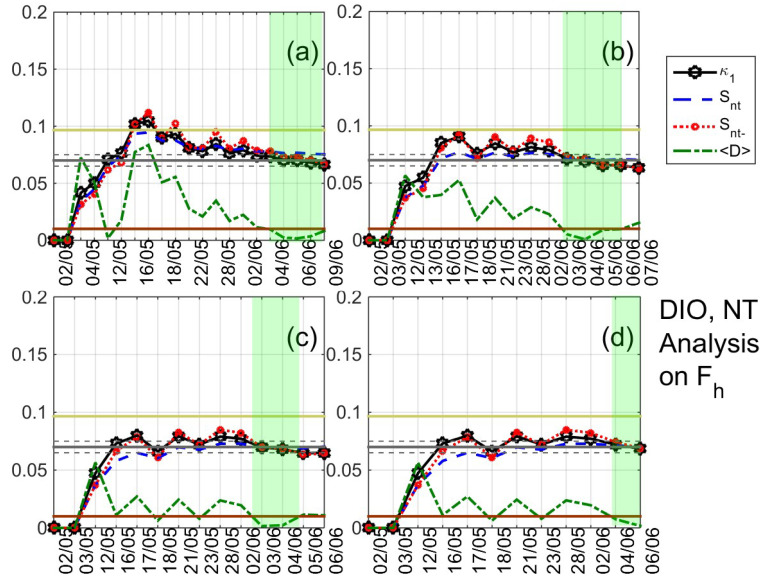
Natural time analysis of $F_{h}$ at DIO: (**a**)–(**d**) Variations of the natural time (NT) analysis parameters ($\kappa_{1}$, $S_{nt}$, $S_{nt-}$, and 〈D〉) for the different thresholds $19\cdot 10^{-5}$, $22.5\cdot 10^{-5}$, $26\cdot 10^{-5}$, and $29.5\cdot 10^{-5}$, respectively. The entropy limit of $S_{u}\left(\approx 0.0966\right)$, the $\kappa_{1}$ value 0.070 and a region of ±0.005 around it are shown by the horizontal solid light green, solid grey, and the grey dashed lines, respectively. Green patches highlight satisfaction of criticality conditions in the parts of the results which are used to reveal true coincidence. Note that the events employed depend on the considered threshold. Moreover, the time (x-) axis is not linear in terms of the conventional time of occurrence of the events, since the employed events appear equally spaced relative to *x*-axis as the NT representation demands, although they are not equally spaced in conventional time. (For the interpretation of the references to colors, the reader is referred to the online version of this paper).

As already mentioned, NT analysis is applied to the daily-valued ULF quantities that are defined in Section 3 [27,28,29,30] for the time period 1 May–12 June 2017, while the criticality criteria are the ones mentioned in Section 4.1 for the case of foreshock seismicity and other cases of limited amount of available data. A typical example of the results that were obtained after the application of NT analysis is shown in Figure 7. It shows the temporal evolution of the NT parameters $\kappa_{1}$, $S_{nt}$, $S_{nt-}$, and 〈D〉 for four threshold values of the ULF quantity $F_{h}$ at DIO. Each time that an $F_{h}$ value exceeds the corresponding threshold, a new event is included in the analysis and all of the studied NT parameters are re-calculated. Green patches indicate the time period during which NT analysis criticality conditions are satisfied for each threshold value. One can observe that, during these periods, $\kappa_{1}$ approaches the value $\kappa_{1}=0.070$ “by descending from above”, $S_{nt},S_{nt-}<S_{u}\left(\approx 0.0966\right)$, and $\langle D\rangle <10^{-2}$, simultaneously. The four time periods are overlapping, intersecting on 4 June 2017, which indicates that critical state is truly achieved on that date.

**Figure 8:**
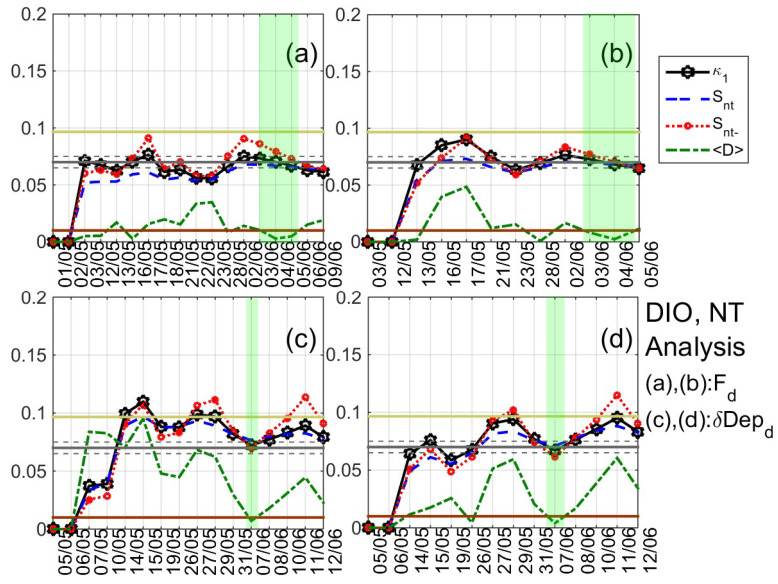
Variations of the NT analysis parameters ($\kappa_{1}$, $S_{nt}$, $S_{nt-}$, and 〈D〉) corresponding to the analysis of $F_{d}$ at DIO for two threshold values $35\cdot 10^{-5}$ (**a**) and $56\cdot 10^{-5}$ (**b**), respectively, as well as of $Dep_{d}$ at the same station for two threshold values 0.1 (**c**) and 0.2 (**d**), respectively. Figure format is similar to Figure 7. (For interpretation of the references to colors, the reader is referred to the online version of this paper).

**Figure 9:**
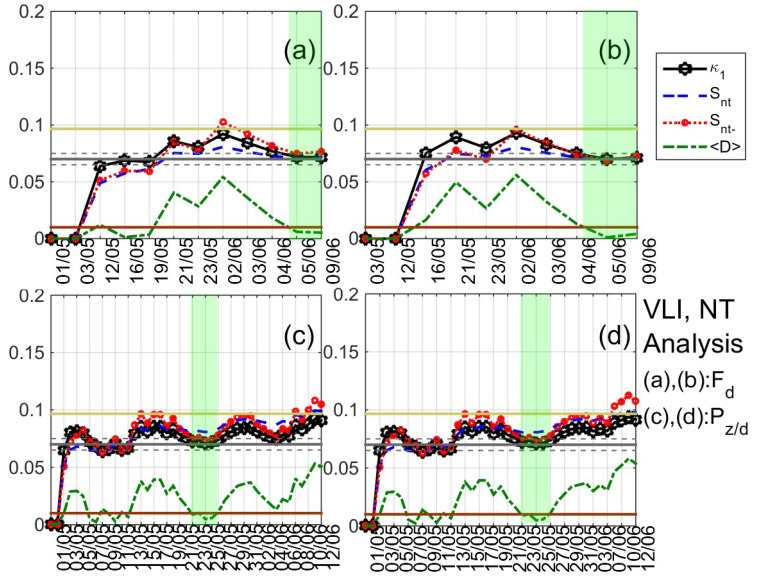
Variations of the NT analysis parameters ($\kappa_{1}$, $S_{nt}$, $S_{nt-}$, and 〈D〉) corresponding to the analysis of $F_{d}$ at VLI for two threshold values $30.245\cdot 10^{-5}$ (**a**) and $41.065\cdot 10^{-5}$ (**b**), respectively, as well as of $P_{z/d}$ at the same station for two threshold values 0.15 (**c**) and 0.26 (**d**), respectively. Figure format is similar to Figure 7. (For the interpretation of the references to colors, the reader is referred to the online version of this paper).

**Figure 10:**
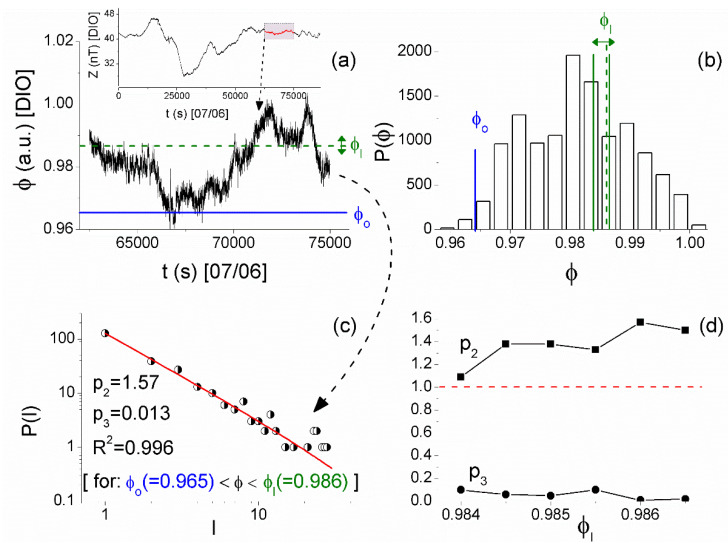
(**a**) The normalized time series excerpt of the Z component of the magnetic field recorded at DIO station of ENIGMA that plays the role of the order parameter ϕ. The inset shows the corresponding part of the original time series recorded on 7 June 2017. (**b**) The histogram of values of ϕ, from which the start of laminar regions $\phi_{o}=0.965$ is determined, while the studied range of ends of laminar regions $\phi_{l}$ is also shown. (**c**) The distribution of the waiting times within the laminar region (laminar lengths) $\phi_{o}\left(=0.965\right)<\phi <\phi_{l}\left(=0.986\right)$ (the specific laminar region is also marked on the analyzed time series by the corresponding horizontal lines shown in (a)). The continuous red line corresponds to the fitted function $f(l)=p_{1}\cdot l^{-p_{2}}\cdot e^{-lp_{3}}$. A clear power-law is identified. (**d**) The exponents $p_{2},p_{3}$ vs. the end point $\phi_{l}$. The validity of criticality condition $p_{2}>1,p_{3}\approx 0$ for a wide range of end point values, and consequently for a wide range of laminar regions, is evident. (For interpretation of the references to colors, the reader is referred to the online version of this paper).

After extensively studying the ULF quantities that are defined in Section 3 for DIO and VLI by means of the NT analysis, signatures of critical dynamics were identified in both stations from 4 June 2017 to 10 June 2017, i.e., from eight to two days before the 2017 Lesvos EQ (indicative results are shown in Figure 8, Figure 9 and Figure 10). In summary,

1. concerning the mean power of horizontal, vertical components and their ratio at DIO, clear criticality indications were found for $F_{h}$ and $F_{d}$ on 4 June 2019 (see Figure 7 and Figure 8a,b), as well as marginal evidence of criticality (just for one threshold value) on 4–7 June 2017 for $P_{z/h}$;

2. concerning the same ULF quantities at VLI, clear criticality indications were found for $F_{d}$ on 5–9 June 2019 (see Figure 9a,b). It is noted that clear criticality indications were also found on 25 May 2019 for $P_{z/d}$ (see Figure 9c,d) and $P_{z/h}$; however, this is more likely to be related with the moderate geomagnetic storm that took place on 28 May 2019 (15 days before the studied EQ);

3. concerning ULF depression at DIO, $\delta Dep_{d}$ presents clear criticality indications on 7 June 2019 (see Figure 8c,d), $Dep_{d}$ presents criticality both on 25 May 2019 (possibly related to the geomagnetic storm) and 10 June 2019 (possibly related to the EQ), while $Dep_{h}$ presents evidence of criticality on 25 May 2019 and on 6 June 2019;

4. concerning ULF depression at VLI, the indications for criticality were found for $\delta Dep_{h}$ on 6 June 2019.

### 4.3. Method of Critical Fluctuations

MCF is based on the theory of phase transitions and the principles of Physics of critical phenomena and starts with the hypothesis of a one-dimensional (1D) non-linear intermittent map of the form $\phi_{n+1}=\phi_{n}+u\phi_{n}^{z}+\epsilon_{n}$ [20,21], where $\phi_{n}$ is the order parameter value at time n, while the shift parameter $\epsilon_{n}$ introduces a non-universal uncorrelated noise that is necessary for the establishment of ergodicity [21]. Note that the corresponding density ρ(ϕ) of such a map resembles, to a large extent, the order parameter distribution close to the critical point, which is characterized by a plateau region and a rapidly decaying tail. By analyzing a time series, supposedly conforming to the above-mentioned map, MCF’s scope is to extract the scaling behavior for the order parameter fluctuations in time, if any, and estimate the critical exponent z (in NT analysis of seismicity, it has been found [39] that the order parameter fluctuations of seismicity are almost simultaneously minimized before major earthquakes [40], with the initiation of SES activities). Notice, that for thermal systems, the exponent z is connected with the isothermal critical exponent δ as z=δ+1. The key idea behind the MCF is that criticality manifests itself by a power-law distribution of properly defined laminar lengths (defined in the following) l, $P(l)\sim l^{-p_{l}}$ [41], where the exponent $p_{l}$ is $p_{l}=1+\frac{1}{\delta }=\frac{z}{z-1}$. More details on the theory behind the method can be found in [20,21,22,23].

In brief, the application of MCF can be described, as follows, while a detailed description of the step-by-step application algorithm can be found in [32]. The analysis starts with a time series excerpt of adequate length (> ~5000 values) presenting, at least, local stationarity, for which the histogram of the order parameter ϕ (usually the original time series amplitude values play the role of the order parameter) is calculated, and from that histogram, an amplitude value (usually on the steepest slope) is determined as the fixed-point $\phi_{o}$, which will serve as the “start of laminar regions”. Following that, for a number of different values within the ϕ amplitude range, which are called “ends of laminar regions” and are denoted as $\phi_{l}$, the distribution P(l) of the “laminar lengths” of each corresponding laminar region $\left(\phi_{o},\phi_{l}\right)$ is calculated (one distribution per $\phi_{l}$ value) and plotted on a log-log plot. Note that laminar lengths are the waiting times within each laminar region $\left(\phi_{o},\phi_{l}\right)$, in other words, the number of successive ϕ-values obeying the condition $\phi_{o}<\phi <\phi_{l}$. Finally, each one of these P(l) distributions is fitted using the function $f(l)=p_{1}\cdot l^{-p_{2}}\cdot e^{-lp_{3}}$, leading to a set of exponents $p_{2},p_{3}$ for each laminar region.

If the exponent $p_{3}$ is zero, then the exponent $p_{2}$ is equal to the exponent $p_{l}$. The relation $p_{l}=\frac{z}{z-1}$ suggests that the exponent $p_{l}$ (or $p_{2}$) should be greater than 1. On the other hand, from the theory of critical phenomena [42], it results that the isothermal exponent δ is greater than 1. Accordingly, from the relation $p_{l}=1+\frac{1}{\delta }$, one obtains the condition $1<p_{l}\left(=p_{2}\right)<2$. In conclusion, the critical profile of the temporal fluctuations is restored for the conditions: $p_{2}>1$ and $p_{3}\approx 0$. As the system departs from the critical state, then the exponent $p_{2}$ decreases, while the exponent $p_{3}$ simultaneously increases, thus reinforcing, in this way, the exponential tail of the laminar lengths distribution. Note that a similar map with opposite signs for u and z, namely $\phi_{n+1}=\phi_{n}-u\phi_{n}^{-z}+\epsilon_{n}$, describes tricritical dynamics [23], which, in terms of MCF analysis, corresponds to the conditions: $p_{2}<1$ and $p_{3}\approx 0$. In the tricritical case, it has been shown that $p_{l}\left(=p_{2}\right)=\frac{z}{z+1}=\frac{\delta +1}{\delta +2}$.

### 4.4. MCF Analysis of ULF Data

As already mentioned, MCF analysis is directly applied to the 1 Hz sampled raw (unfiltered) magnetometer measurements [31,32]. An example of the results that were obtained during the successive steps of the application of MCF is shown in Figure 10. It refers to part of the Z component recordings of the magnetic field at DIO (see inset of Figure 10a) that was found to be stationary by checking its cumulative mean value and the corresponding standard deviation using nested time series excerpts of progressively wider length. By applying the MCF algorithm that is presented in detail in [32], it was found that it is necessary to add uniform noise to the original time series (after normalizing it in the range [0,1]) for the establishment of ergodicity [21]. Specifically, uniform noise within the interval [−0.002,0.002] was added and the resultant time series (Figure 10a) was considered to play the role of the order parameter in further applying MCF. From the histogram of this time series amplitude values (Figure 10b), the fixed point (start of laminar regions) $\phi_{o}=0.965$ was determined according to the turning point method [43,44]. Subsequently, for a number of different ends of laminar regions, $\phi_{l}$, (see Figure 10b and Figure 10a) the waiting times within each laminar region were calculated, and subsequently the distributions P(l) of these waiting times (laminar lengths) were plotted and fitted by the function $f(l)=p_{1}\cdot l^{-p_{2}}\cdot e^{-lp_{3}}$. Figure 10c shows one such example for the laminar region $\phi_{o}\left(=0.965\right)<\phi <\phi_{l}\left(=0.986\right)$, while Figure 10d shows the exponents $p_{2},p_{3}$ that were obtained for the different laminar regions. From Figure 10c,d, one can see that the MCF criticality condition $p_{2}>1,p_{3}\approx 0$ is valid for a wide range of end point values, denoting that the distribution P(l) of laminar lengths follows a power law.

**Figure 11:**
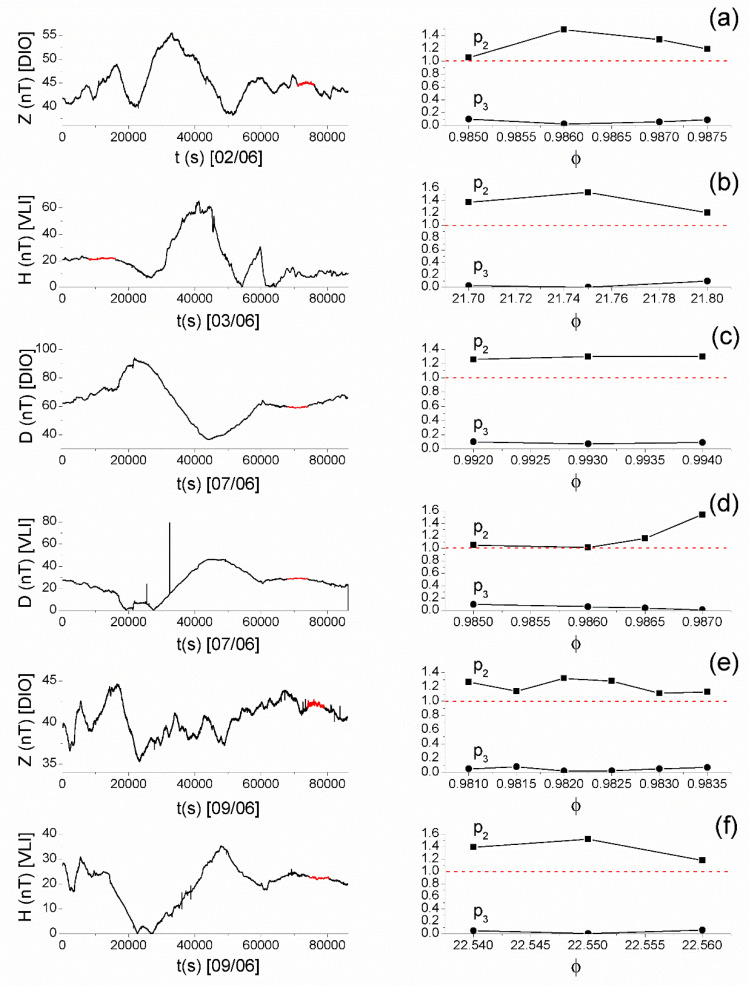
Left panels show the recordings of specific days at specific stations of the ENIGMA array, while right panels show the corresponding results obtained after the method of critical fluctuations (MCF) analysis of parts of the recordings (shown in red color on the total recordings) that were found to satisfy MCF criticality conditions: (**a**) Z component* of DIO on 2 June 2017, (**b**) H component of VLI on 3 June 2017, (**c**) D component* of DIO on 7 June 2017, (**d**) D component* of VLI on 7 June 2017, (**e**) Z component* of DIO on 9 June 2017, and (**f**) H component of VLI on 9 June 2017. (For interpretation of the references to colors, the reader is referred to the online version of this paper.). (* = For the specific recordings it was found necessary to add uniform noise in the range $\left[-\varepsilon_{0},\varepsilon_{0}\right]$, with $\varepsilon_{0}$ of the order of $10^{-2}$, after normalizing the analyzed time series excerpt in the range [0,1], for the establishment of ergodicity that is necessary for the application of MCF [21]).

After extensively studying the magnetic field recordings at DIO and VLI by means of the MCF, the signatures of critical dynamics were identified in both stations from 2 June 2017 to 9 June 2017. Specifically,

1. on 2 June 2017 in the Z component recordings of DIO from 19:43:20 UT to 21:06:40 UT (Figure 11a)

2. on 3 June 2017 in the H component of VLI from 02:13:20 UT to 04:26:40 UT (Figure 11b)

3. on 7 June 2017 in the Z component of DIO (Figure 10), as well as in the D components of DIO and VLI (Figure 11c,d) practically during the same time window (17:21:40–20:50:00, 18:36:40–20:33:20, and 18:53:20–20:33:20 UT, respectively)

4. on 9 June 2017 in the Z component of DIO (20:33:20–21:56:40) UT (Figure 11e) and the H component of VLI (20:41:40–22:21:40) UT (Figure 11f), also largely during overlapping time windows.

## 5. Possible Fracto-EME EQ Precursors

**Figure 12:**
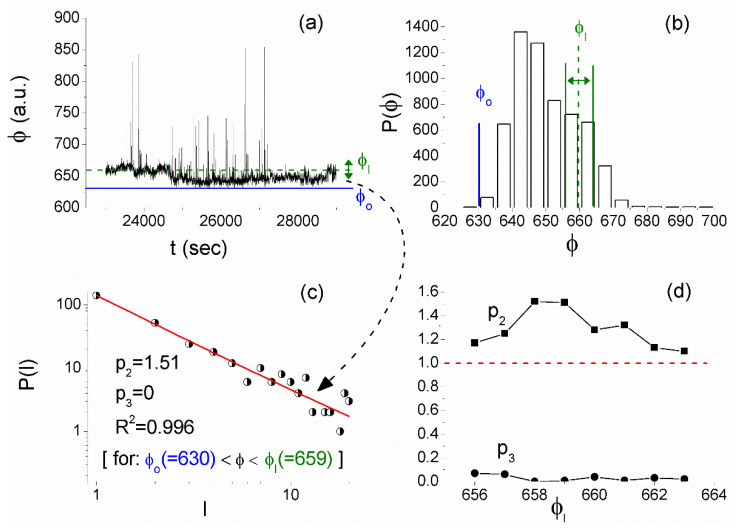
MCF analysis of the 41 MHz fracto-EME recorded on 01/06/2017 (06:23:20–08:03:20) UT at the station M of ELSEM-Net. (**a**) The amplitude of the acquired time series plays the role of the order parameter ϕ (time in (s) from 1 June 2017 00:00:00 UT). (**b**) The histogram of values of ϕ from which the start of laminar regions $\phi_{o}=630$ is determined, while the studied range of ends of laminar regions $\phi_{l}$ is also shown. (**c**) The distribution of the waiting times within the laminar region (laminar lengths) $\phi_{o}\left(=630\right)<\phi <\phi_{l}\left(=659\right)$ (the specific laminar region is also marked on the analyzed time series by the corresponding horizontal lines shown in (a)). The continuous red line corresponds to the fitted function $f(l)=p_{1}\cdot l^{-p_{2}}\cdot e^{-lp_{3}}$. A clear power-law is identified. (**d**) The exponents $p_{2},p_{3}$ vs. the end point $\phi_{l}$. The validity of criticality condition $p_{2}>1,p_{3}\approx 0$ for a wide range of end point values, and consequently for a wide range of laminar regions, is evident. (For interpretation of the references to colors, the reader is referred to the online version of this paper).

**Figure 13:**
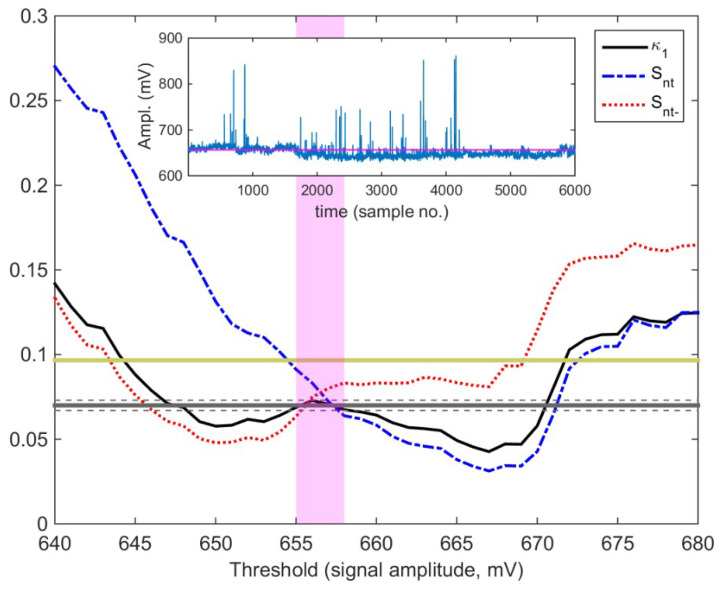
The NT analysis quantities $\kappa_{1}$ (solid curve), $S_{nt}$ (dash-dot curve), and $S_{nt-}$ (dot curve) vs. amplitude threshold for the 41 MHz signal of Figure 12a (also shown in the inset). The entropy limit of $S_{u}\left(\approx 0.0966\right)$, the value 0.070 and a region of ±0.005 around it are denoted by the horizontal solid light green, solid grey, and the grey dashed lines, respectively. The vertical orthogonal magenta shaded area indicates the thresholds range for which criticality conditions according to the NT analysis method are satisfied. (For interpretation of the references to colors, the reader is referred to the online version of this paper).

**Figure 14:**
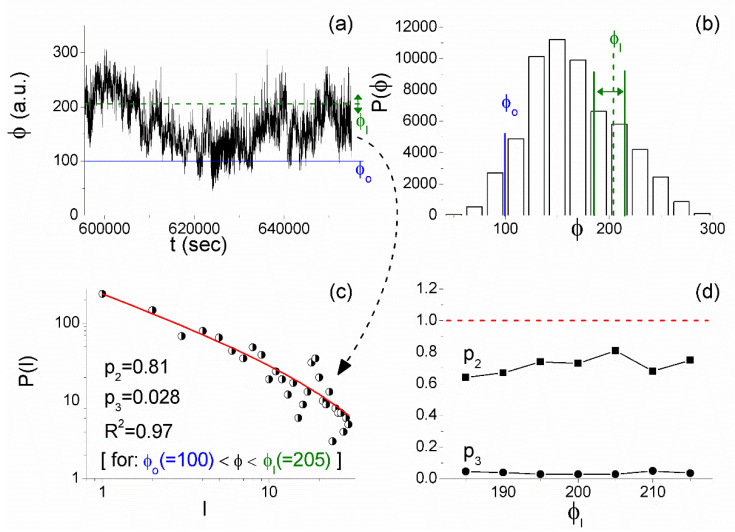
MCF analysis of the 3 kHz east–west fracto-EME recorded between 5 June 2017 21:25:55 UT and 6 June 2017 14:05:55 UT at the station M of ELSEM-Net. (**a**) The amplitude of the acquired time series plays the role of the order parameter ϕ (time in (s) from 30 May 2017 00:00:00 UT). (**b**) The histogram of values of ϕ from which the start of laminar regions $\phi_{o}=100$ is determined, while the studied range of ends of laminar regions $\phi_{l}$ is also shown. (**c**) The distribution of the waiting times within the laminar region (laminar lengths) $\phi_{o}\left(=100\right)<\phi <\phi_{l}\left(=205\right)$ (the specific laminar region is also marked on the analyzed time series by the corresponding horizontal lines shown in (**a**)). The continuous red line corresponds to the fitted function $f(l)=p_{1}\cdot l^{-p_{2}}\cdot e^{-lp_{3}}$. A clear power-law is identified. (**d**) The exponents $p_{2},p_{3}$ vs. the end point $\phi_{l}$. The validity of tricriticality condition $p_{2}<1,p_{3}\approx 0$ for a wide range of end point values, and consequently for a wide range of laminar regions, is evident. (For interpretation of the references to colors, the reader is referred to the online version of this paper).

In parallel with the analysis of the magnetic field recordings of the ENIGMA array of magnetometers, the MHz and kHz fracto-EME recordings of the stations of the ELSEM-Net network were also analyzed in the search of possible EQ precursors. After the analysis of both MHz and kHz recordings at different stations, using multiple time series analysis tools, it was found that (preliminary results have been presented in the pre-publication [45]):

1. Critical signature was identified in the 41 MHz recordings of station M (located on the Island of Lesvos). Specifically, a ~1.7 h long time window (06:23:20–08:03:20) UT on 1 June 2017 was found by MCF (Figure 12) and NT analysis (Figure 13) to present critical characteristics.

2. A few days after the appearance of the criticality in the 41 MHz recordings, tricritical behavior was found by means of MCF in the kHz fracto-EME recordings of the same station (both at the 3 kHz and 10 kHz) from 5 June 2017 21:25:55 UT–6 June 2017 14:05:55 UT (~16.5 h long time window). An example for 3 kHz east–west is shown in Figure 14.

3. No very strong, avalanche like, precursory kHz fracto-EME signals of clearly high organization and persistency were possible to be validated (by means of different entropic/information metrics, Hurst exponent, detrended fluctuation analysis (DFA), spectral exponent analysis, fractal dimension etc.). This is probably due to the fact that the EQ of interest happened in the Sea as it is discussed in the following.

From Figure 12d, one can verify that the MCF criticality condition $p_{2}>1,p_{3}\approx 0$ is satisfied for a wide range of laminar regions, while from Figure 14d, it is obvious that the tricriticality conditions $p_{2}<1$ and $p_{3}\approx 0$ are satisfied for a wide range of end points $\phi_{l}$. It is noted that MCF analysis was performed in a similar way to the one that is presented in Section 4.4 for the ULF magnetic field recordings, e.g., Figure 10.

On the other hand, NT analysis, as already mentioned in Section 4.1, was not performed in the same way as the NT analysis of the daily-valued ULF quantities that were analyzed in Section 4.2. In the MHz fracto-EME case, the criticality criteria according to NT analysis are that parameter $\kappa_{1}$ converges to the value $\kappa_{1}=0.070$, and at the same time, both the entropy in NT, $S_{nt}$, and the entropy under time reversal, $S_{nt-}$, satisfy the condition $S_{nt},S_{nt-}<\left(\ln2/2\right)-1/4$ [8,9,10].

It should be noted that the MHz fracto-EME time series in most cases are not in the form of clearly distinguishable bursts. Therefore, there is not an easy way to define the involved fracto-EME events (the energy of which is attributed to the important NT analysis quantity $Q_{k}$) [8], because this involves the determination of a threshold value, corresponding to the background noise level, above which the amplitude is taken into account for the calculations. In this case, it has been proposed [8] that an exhaustive search should be performed for the determination of the threshold value. This way, one can exclude thresholds that may not lead to reliable $\kappa_{1}$ values, because of possible contamination by uneliminated noise [8]. The key idea behind this approach is that, if the time series excerpt under analysis presents criticality characteristics, then there should be at least one threshold value for which the above-mentioned NT criticality conditions are satisfied. If there is no such threshold, and then the specific excerpt does not present criticality [8].

From Figure 13, one can verify that for the threshold range marked by the vertical orthogonal magenta shaded area the NT analysis criticality conditions are satisfied, since $\kappa_{1}$ converges to $\kappa_{1}=0.070$, and at the same time, both $S_{nt}$ and $S_{nt-}$ satisfy the condition $S_{nt},S_{nt-}<\left(\ln2/2\right)-1/4\left(\approx 0.0966\right)$.

Our analysis reveals that first in the timeline appear critical features in the MHz fracto-EME, implying that the possibly related underlying fracture process that is involved in the preparation of the main shock is at critical state. The presence of the “critical point” during which any two active parts of the system are highly correlated, even at arbitrarily long distances, in other words, when “everything depends on everything else”, is consistent with the view that the EQ preparation process during the period that the MHz fracto-EME precursory signals are emitted is a spatially extensive process. It is noted that, according to the four-stage model of EQ dynamics by means of fracto-EME, as suggested in [7], the pre-seismic critical MHz fracto-EME, which corresponds to the first stage of the specific model, is considered to originate during the fracture of the part of the Earth’s crust that is characterized by high heterogeneity. During this phase, the fracture is non-directional and it spans over a large area that surrounds the family of large high-strength entities (asperities) that are distributed along the main fault sustaining the system. Note that, for an EQ of magnitude ~6.5, such as the 2017 Lesvos EQ of interest, the corresponding fracture process extends to a critical radius of ~120 km [46]. Thus, during this phase, the fracture process is extended up to the land of the neighboring islands. For the EQ of interest, the analysis also reveals that next in the timeline, after the critical MHz fracto-EME, appear tricritical fracto-EME (kHz in the specific case). These correspond to the second stage of the aforementioned model. Tricritical dynamics signify the departure from critical state, implying that the possibly related underlying fracture process that is involved in the preparation of the main shock evolves from the highly symmetrical and spatially expanded phase to a low symmetry, spatially focused on a preferred direction (along the fault) phase [10,23]. The observed EM silence that follows up to the EQ occurrence corresponds to the fourth stage of the aforementioned model.

As already mentioned, the crucial very strong avalanche, like precursory kHz fracto-EME signals, which are recorded in the tail of the preseismic kHz EM emission in the case of shallow EQs that happen inland (or near coast-line) and correspond to the third stage of the aforementioned four-stage model, were not recorded prior to the seismic event under study. It has been suggested that the lounge of the kHz EM activity shows the fracture of asperities sustaining the main fault that corresponds to the third stage of the four-stage model. In the case under study, the fracture process during this stage has been confined to a narrow area, i.e., along the main fault in a submerged area. This situation by itself justifies the observed absence. On the other hand, in terms of percolation theory, the “hydraulic threshold”, $x_{c}$, during which the transition impermeable–permeable occurs, as well as the “mechanical” or “damage threshold”, $x_{m}\left(x_{m}>x_{c}\right)$, during which the infinite cluster (IC) is formed and the solid disintegrates, precede the shear displacement along the fault plane (the EQ), which means that many transport properties are activated before the main event [47], and references therein. This fact, when combined with the governing role of local hydraulic conditions on the water injection-induced fracture behavior in rocks concerning the mechanical properties, fracture nucleation, and the geometry of the shear fracture zone [47], further enhances the absence of the final strong pulse, like kHz fracto-EME. We note that, due to the crucial character of this emission, an austere set of criteria have been established to characterize such a recorded kHz anomaly as a seismogenic one [7,48,49]. The multidisciplinary analysis of the kHz records before the under study seismic event in terms of these criteria verified the absence of the last emerged part of the fracture induced kHz fracto-EME corresponding to the fracture of asperities.

## 6. Summary and Conclusions

This paper probably reports the first use of ULF magnetic field data from a space weather monitoring magnetometer array in Greece in the study of seismo-electromagnetics or EQ precursor studies. Our target was a rather big EQ occurred on 12 June 2017 at Lesvos Island in Greece with magnitude of 6.3.

First, the data from four stations of the Greek ULF network ENIGMA have been examined, and the detailed analyses that are based on the conventional statistical method have been performed for a time period from 1 May to 17 June 2017. We can summarize the obtained observational results on the precursors to this EQ as follows:

(1) In order to find any ULF precursors, we found that the reliable time interval was nighttime as usual and the most important frequency range was 20–30 mHz (0.02–0.03 Hz), as already confirmed before [13,14,15,16,18].

(2) The most reliable and conspicuous precursor was ULF depression (most enhanced depression of the horizontal components of magnetospheric ULF waves), which was detected one day before the EQ. The more reliable results were obtained at a farther station of VLI (distance about 400 km). This kind of high sensitivity of the ULF depression phenomenon provides a further confirmation of our previous findings [15,16,17] and this ULF depression is likely to be attributed to the perturbation of the lower ionosphere [17].

(3) The direct ULF emission from the lithosphere as the result EQ preparation process is likely to be detected at the closest station of DIO (distance ~250 km from EQ epicenter) in the form of enhanced in $P_{z/d}$ and $P_{z/h}$. This effect is also observed, albeit not so clearly, at the more distant station of VLI (distance ~400 km from EQ epicenter) and it does not appear in the rest two distant stations THL and FIN. This ULF lithospheric emission is known to be a much more local effect than the above ULF depression. The ULF lithospheric emission appearing at DIO four days before the EQ is convincing, but that appearing one day before the EQ is not so reliable because it might be related with the above ULF depression one day before the EQ. The lead time of four days seems to be much smaller than the conventional lead time of 2–3 weeks [13,14].

(4) The geomagnetic activity during the whole period of analysis seems to be rather quiet; that is, the maximum geomagnetic activity appeared in the end of May and the geomagnetic activity was quiet when the above precursors were detected.

Based on these results, we further investigated the ULF magnetic field data by applying criticality analysis for the two stations (DIO and VLI) for which possible precursors to the EQ were identified. The results that were obtained by two independent criticality analysis methods, NT analysis and MCF, revealed several indications of critical dynamics eight to two days and ten to three days before the EQ, respectively. Specifically:

(1) Clear criticality indications were identified in the mean power of the horizontal components of the magnetic field (ULF quantities $F_{h}$ and $F_{d}$) on 4 June 2019 at DIO and on 5–9 June 2019 (in $F_{d}$) at VLI by means of NT analysis. While marginal evidence of criticality were found on 4–7 June 2017 for $P_{z/h}$ at DIO.

(2) Clear criticality indications were also identified in terms of NT analysis in the ULF depression quantities $\delta Dep_{d}$ on 07/06/2019, $Dep_{d}$ on 10 June 2019, and $Dep_{h}$ on 6 June 2019 at DIO, while $\delta Dep_{h}$ at VLI presents criticality on 6 June 2019.

(3) NT analysis also identified criticality indications on 25 May 2017 in $P_{z/d}$ and $P_{z/h}$ at VLI, as well as in $Dep_{d}$ and $Dep_{h}$ at DIO. However, this is more likely to be related with the moderate geomagnetic storm that took place on 28 May 2019 (15 days before the studied EQ).

(4) MCF analysis revealed criticality on 2 June 2017 in the Z component of DIO and on 3 June 2017 in the H component of VLI

(5) Practically simultaneous evidence of critical dynamics were found by means of MCF on 7 June 2017 in the Z component of DIO, in the D component of DIO, and D component of VLI.

(6) The same was found for the fluctuations of the Z component of DIO and the H component of VLI on 9 June 2017.

In order to find out whether other EM effects that were possibly related to the specific EQ took place, the data from the ground-based fracto-EME station network ELSEM-Net spanning across Greece have been analyzed, leading to the following results:

(1) The MHz recordings of 1 June 2017 (11 days before the EQ) at the station located in Lesvos Island, very close to the EQ epicenter, presented criticality characteristics (by means of both NT analysis and MCF).

(2) A few days later, on 5 June 2017 (7–6 days before the EQ), the kHz recordings of the same station presented tricritical behavior by means of MCF.

(3) No very strong, avalanche like, precursory kHz fracto-EME signals of clearly high organization and persistency was possible to be validated (by means of different entropic/information metrics, Hurst exponent, DFA, spectral exponent analysis, fractal dimension, etc.). This is probably due to the fact that the 2017 Lesvos EQ happened under the Sea, as discussed in Section 5.

The above summarized results suggest that three types of possible precursory signs appeared before the 2017 Lesvos EQ: (a) noticeable change in the mean power of appropriately defined ULF magnetic field quantities that are possibly related to either direct lithospheric emissions or ionospheric depression, (b) approach to critical state for some of the abovementioned quantities as well as for raw magnetic field measurements and MHz fracto-EME, and (c) tricritical dynamics for kHz fracto-EME.

We note that the criticality indications that have been identified for the different EM signals all appear a few days before EQ occurrence. The EQ preparation process has various facets that reflect corresponding different EM signals. Indifferent to the mechanisms by which they are produced, the EQ-related MHz fracto-EME, direct lithospheric ULF emissions, and ionospheric ULF depression seem to be compatible with each other in terms of criticality. These EM precursors emerge during a “critical epoch”, when the “short-range” correlations evolve into “long-range” ones, suggesting a spatially extensive process for their generation. For example, the generation of a preseismic ionospheric anomaly requires physical and chemical transformations that occur in a spatially extended preparation (activation) zone of an impending EQ. Such a requirement is satisfied during the appearance of the “critical epoch”.

The obtained results show that the ULF magnetic field data of the ENIGMA magnetometer array, beyond their primary use for the study of space weather and extreme space weather events, such as magnetic storms [50], can be efficiently employed for the detection of possible precursors of seismic activity by applying advanced analysis methods. Moreover, the fusion of the information obtained from these ULF magnetic field data with information from other kinds of EM data, such as subionospheric VLF/LF (low frequency) propagation data (ionospheric perturbations), ionosonde data, satellite data, fracto-EME (MHz, kHz) signals, SES signals, etc., but also gas emanation data, such as radon concentrations in boreholes, as well as tectonic information, seismic zonation, and foreshock seismic activity data, may: (a) help deciphering the physical mechanisms that are related to different seismo-electromagnetic phenomena (e.g., elucidating the LAI coupling mechanism) and (b) contribute to a future multi-parametric seismic risk assessment system.

## Figures and Tables

**Table 1:** ENIGMA magnetometer stations information.

No	Station Code	Location	Latitude (°N)	Longitude (°E)	Altitude (m)	Instrumentation	Sampling Frequency
**1**	THL	Klokotos, Trikala	39.5646	22.0144	86	GEOMAG-02 magnetotelluric station	5 Hz
**2**	DIO	Dionysos, Attica	38.0779	23.9331	460	GEOMAG-02M fluxgate magnetometer	1 Hz
**3**	VLI	Velies, Lakonia	36.7180	22.9468	220	GEOMAG-02 magnetotelluric station	1 Hz
**4**	FIN	Finokalia, Crete Island	35.338	25.670	233	GEOMAG-02 magnetotelluric station	10 Hz

**Table 2:** Location of the fracto-EME stations of ELSEM-Net. (* = Currently non-operating).

Νο	Station Code	Location	Latitude (^o^ N)	Longitude (^o^ E)	Altitude (m)
**1**	J	Ioannina	39.6561	20.8487	526
**2**	H	Atalandi	38.6495	22.9988	185
**3**	F	Valsamata, Cephalonia Island	38.1768	20.5886	402
**4**	O	Ithomi, Mesinia	37.1787	21.9252	423
**5**	K	Kozani	40.3033	21.7820	791
**6**	E	Neapoli, Crete Island	35.2613	25.6103	288
**7**	V	Vamos, Crete Island	35.4070	24.1997	225
**8**	A	Archangelos, Rhodes Island	36.2135	28.1212	148
**9**	T	Komotini	41.1450	25.5355	116
**10**	M	Agia Paraskevi, Lesvos Island	39.2456	26.2649	130
**11**	Z (*)	Fterini-Agios Leon, Zakynthos Island	37.7658	20.7430	461

## References

[1] Pulinets S.A., Boyarchuk K. (2004). Ionospheric Precursors of Earthquakes.

[2] Molchanov O.A., Hayakawa M. (2008). Seismo Electromagnetics and Related Phenomena: History and Latest Results.

[3] Hayakawa M. (2015). Earthquake Prediction with Radio Techniques.

[4] Ouzounov D., Pulinets S., Hattori K., Taylor P. (2018). Pre-earthquake Processes: A Multidisciplinary Approach to Earthquake Prediction Studies.

[5] Varotsos P.A. (2005). The Physics of Seismic Electric Signals.

[6] Sarlis N.V., Skordas E.S. (2018). Study in natural time of geoelectric field and seismicity changes preceding the Mw6.8 earthquake on 25 October 2018 Greece. Entropy.

[7] Eftaxias K., Potirakis S.M., Contoyiannis Y., Chelidze T., Vallianatos F., Telesca L. (2018). Four-stage model of earthquake generation in terms of fracture-induced electromagnetic emissions. Complexity of Seismic Time Series: Measurement and Application.

[8] Potirakis S.M., Karadimitrakis A., Eftaxias K. (2013). Natural time analysis of critical phenomena: The case of pre-fracture electromagnetic emissions. Chaos.

[9] Potirakis S.M., Contoyiannis Y., Eftaxias K., Koulouras G., Nomicos C. (2015). Recent field observations indicating an earth system in critical condition before the occurrence of a significant earthquake. IEEE Geosci. Remote Sens. Lett..

[10] Potirakis S.M., Contoyiannis Y., Melis N.S., Kopanas J., Antonopoulos G., Balasis G., Kontoes C., Nomicos C., Eftaxias K. (2016). Recent seismic activity at Cephalonia (Greece): A study through candidate electromagnetic precursors in terms of nonlinear dynamics. Nonlinear Process. Geophys..

[11] Varotsos P.A., Sarlis N.V., Skordas E.S. (2003). Electric fields that ‘‘arrive’’ before the time derivative of the magnetic field prior to major earthquakes. Phys. Rev. Lett..

[12] Kopytenko Y.A., Matiashvily T.G., Voronov P.M., Kopytenko E.A., Molchanov O. (1990). Detection of ULF emission connected with the Spitak earthquake and its aftershock activity based on geomagnetic pulsations data at Dusheti and Vardziya observatories. Phys. Earth Planet. Inter..

[13] Hayakawa M., Hobara Y., Ohta K., Hattori K. (2011). The ultra-low-frequency magnetic disturbances associated with earthquakes. Earthq. Sci..

[14] Hattori K., Hayakawa M. (2013). ULF geomagnetic changes associated with major earthquakes. Earthquake Prediction Studies: Seismo Elecromagnetics.

[15] Schekotov A., Molchanov O., Hattori K., Fedorov E., Gradyshev V.A., Belyaev G.G., Chebrov V., Sinitsin V., Gordeev E., Hayakawa M. (2006). Seismo-ionospheric depression of the ULF geomagnetic fluctuations at Kamchatka and Japan. Phys. Chem. Earth.

[16] Schekotov A., Fedorov E., Molchanov O.A., Hayakawa M., Hayakawa M. (2013). Low frequency electromagnetic precursors as a prospect for earthquake prediction. Earthquake Prediction Studies: Seismo Electromagnetics.

[17] Schekotov A., Hayakawa M. (2017). ULF/ELF Electromagnetic Phenomena for Short-term Earthquake Prediction.

[18] Hayakawa M., Kawate R., Molchanov O.A., Yumoto K. (1996). Results of ultra-low-frequency magnetic field measurements during the Guam earthquake of 8 August 1993. Geophys. Res. Lett..

[19] Varotsos P.A., Sarlis N.V., Skordas E.S. (2011). Natural Time Analysis: The New View of Time.

[20] Contoyiannis Y., Diakonos F. (2000). Criticality and intermittency in the order parameter space. Phys. Lett. A..

[21] Contoyiannis Y.F., Diakonos F.K. (2007). Unimodal maps and order parameter fluctuations in the critical region. Phys. Rev. E..

[22] Contoyiannis Y., Diakonos F., Malakis A. (2002). Intermittent dynamics of critical fluctuations. Phys. Rev. Lett..

[23] Contoyiannis Y., Potirakis S.M., Eftaxias K., Contoyianni L. (2015). Tricritical crossover in earthquake preparation by analyzing preseismic electromagnetic emissions. J. Geodynamics..

[24] Gjerloev J.W. (2009). A global ground-based magnetometer initiative. EOS..

[25] Hayakawa M. (2011). Probing the lower ionospheric perturbations associated with earthquakes by means of subionospheric VLF/LF propagation. Earthq. Sci..

[26] Hayakawa M., Asano T., Rozhnoi A., Solovieva M., Ouzounov D., Pulinets S., Hattori K., Taylor P. (2018). Very-low- and low-frequency sounding of ionospheric perturbations and possible association with earthquakes. Pre-earthquake Processes: A Multidisciplinary Approach to Earthquake Prediction Studies.

[27] Hayakawa M., Schekotov A., Potirakis S., Eftaxias K. (2015). Criticality features in ULF magnetic fields prior to the 2011 Tohoku earthquake. Proc. Jpn. Acad. Ser. B.

[28] Hayakawa M., Schekotov A., Potirakis S.M., Eftaxias K., Li Q., Asano T. (2015). An integrated study of ULF magnetic field variations in association with the 2008 Sichuan earthquake, on the basis of statistical and critical analyses. Open J. Earthq. Res..

[29] Potirakis S.M., Eftaxias K., Schekotov A., Yamaguchi H., Hayakawa M. (2016). Criticality features in ultra-low frequency magnetic fields prior to the 2013 M6.3 Kobe earthquake. Ann. Geophys..

[30] Potirakis S.M., Schekotov A., Asano T., Hayakawa M. (2018). Natural time analysis on the ultra-low frequency magnetic field variations prior to the 2016 Kumamoto (Japan) earthquakes. J. Asian Earth Sci..

[31] Contoyiannis Y., Potirakis S.M., Eftaxias K., Hayakawa M., Schekotov A. (2016). Intermittent criticality revealed in ULF magnetic fields prior to the 11 March 2011 Tohoku earthquake (Mw = 9). Physica A..

[32] Potirakis S.M., Contoyiannis Y., Schekotov A., Asano T., Hayakawa M. (2019). Analysis of the ultra-low frequency magnetic field fluctuations prior to the 2016 Kumamoto (Japan) earthquakes in terms of the method of critical fluctuations. Physica A.

[33] Varotsos P.A., Sarlis N.V., Skordas E.S., Tanaka H.K., Lazaridou M.S. (2006). Entropy of seismic electric signals: Analysis in the natural time under time reversal. Phys. Rev. E.

[34] Varotsos P.A., Sarlis N.V., Skordas E.S. (2001). Spatio-temporal complexity aspects on the interrelation between seismic electric signals and seismicity. Pract. Athens Acad..

[35] Varotsos P.A., Sarlis N.V., Skordas E.S. (2002). Long-range correlations in the electric signals that precede rupture. Phys. Rev. E.

[36] Varotsos P.A., Sarlis N.V., Tanaka H.K., Skordas E.S. (2005). Similarity of fluctuations in correlated systems: The case of seismicity. Phys. Rev. E.

[37] Potirakis S.M., Asano T., Hayakawa M. (2018). Critical analysis of the lower ionosphere perturbations prior to the 2016 Kumamoto (Japan) earthquakes as based on VLF electromagnetic wave propagation data observed at multiple stations. Entropy.

[38] Sarlis N.V., Skordas E.S., Lazaridou M.S., Varotsos P.A. (2008). Investigation of seismicity after the initiation of a Seismic Electric Signal activity until the main shock. Proc. Jpn Acad. Ser. B.

[39] Sarlis N.V., Skordas E.S., Varotsos P.A., Nagao T., Kamogawa M., Tanaka H., Uyeda S. (2013). Minimum of the order parameter fluctuations of seismicity before major earthquakes in Japan. Proc. Natl. Acad. Sci. USA.

[40] Varotsos P.A., Sarlis N.V., Skordas E.S., Lazaridou M.S. (2013). Seismic Electric Signals: An additional fact showing their physical interconnection with seismicity. Tectonophysics.

[41] Schuster H. (1998). Deterministic Chaos.

[42] Huang K. (1987). Statistical Mechanics, 2nd Edition.

[43] Diakonos F.K., Schmelcher P. (1997). Turning point properties as a method for the characterization of the ergodic dynamics of one- dimensional iterative maps. Chaos.

[44] Schmelcher P., Diakonos F.K. (1997). A turning point analysis of the ergodic dynamics of iterative maps. Int. J. Bifurcation Chaos.

[45] Contoyiannis Y., Potirakis S.M., Kopanas J., Antonopoulos G., Koulouras G., Eftaxias K., Nomicos C. On the recent seismic activity at eastern Aegean Sea: Analysis of fracture-induced electromagnetic emissions in terms of critical fluctuations. https://arxiv.org/abs/1708.00320.

[46] Bowman D., Quillon G., Sammis C., Sornette A., Sornette D. (1998). An observational test of the critical earthquake concept. J. Geophys. Res..

[47] Li X., Lei X., Li Q. (2016). Injection-induced fracturing process in a tight sandstone under different saturation conditions. Environ. Earth Sci..

[48] Eftaxias K., Potirakis S.M. (2013). Current challenges for pre-earthquake electromagnetic emissions: Shedding light from micro-scale plastic flow, granular packings, phase transitions and self-affinity notion of fracture process. Nonlin. Process. Geophys..

[49] Eftaxias K., Potirakis S.M., Chelidze T. (2013). On the puzzling feature of the silence of precursory electromagnetic emissions. Nat. Hazards Earth Syst. Sci..

[50] Balasis G., Daglis I.A., Contoyiannis Y., Potirakis S.M., Papadimitriou C., Melis N.S., GIannakis O., Papaioannou A., Anastasiadis A., Kontoes C. (2018). Observation of Intermittency-Induced Critical Dynamics in Geomagnetic Field Time Series Prior to the Intense Magnetic Storms of March, June, and December 2015. J. Geophys. Res. Space Phys..

